# CIRF: Coupled Image Reconstruction and Fusion Strategy for Deep Learning Based Multi-Modal Image Fusion

**DOI:** 10.3390/s24113545

**Published:** 2024-05-30

**Authors:** Junze Zheng, Junyan Xiao, Yaowei Wang, Xuming Zhang

**Affiliations:** Department of Biomedical Engineering, College of Life Science and Technology, Huazhong University of Science and Technology, Wuhan 430074, China

**Keywords:** multi-modal medical image fusion, multi-task learning, deep learning, vision transformer, convolutional neural network

## Abstract

Multi-modal medical image fusion (MMIF) is crucial for disease diagnosis and treatment because the images reconstructed from signals collected by different sensors can provide complementary information. In recent years, deep learning (DL) based methods have been widely used in MMIF. However, these methods often adopt a serial fusion strategy without feature decomposition, causing error accumulation and confusion of characteristics across different scales. To address these issues, we have proposed the Coupled Image Reconstruction and Fusion (CIRF) strategy. Our method parallels the image fusion and reconstruction branches which are linked by a common encoder. Firstly, CIRF uses the lightweight encoder to extract base and detail features, respectively, through the Vision Transformer (ViT) and the Convolutional Neural Network (CNN) branches, where the two branches interact to supplement information. Then, two types of features are fused separately via different blocks and finally decoded into fusion results. In the loss function, both the supervised loss from the reconstruction branch and the unsupervised loss from the fusion branch are included. As a whole, CIRF increases its expressivity by adding multi-task learning and feature decomposition. Additionally, we have also explored the impact of image masking on the network’s feature extraction ability and validated the generalization capability of the model. Through experiments on three datasets, it has been demonstrated both subjectively and objectively, that the images fused by CIRF exhibit appropriate brightness and smooth edge transition with more competitive evaluation metrics than those fused by several other traditional and DL-based methods.

## 1. Introduction

With the development of medical imaging technology, a variety of imaging modalities have emerged, such as magnetic resonance imaging (MRI) [1], computed tomography (CT) [2], positron emission tomography (PET) [3] and single-photon emission computed tomography (SPECT) [4]. They all have unique information and characteristics [5]. MR images have better soft tissue definition and higher spatial resolution but are often accompanied by motion artifacts. CT images can facilitate the detection of dense structures like bones and implants; however, CT imaging involves a certain level of radiation and is limited in its ability to provide qualitative diagnosis. PET and SPECT images have high sensitivity and are often used for metabolic information gauging, vascular disease diagnosis and tumor detection, but their spatial resolution is relatively low.

From the above discussion, it is clear that each imaging modality has its own scope of application and limitations. Furthermore, information from a single sensor is not enough to handle scene changes effectively, and the information from different modalities is exceedingly significant. Additionally, even when there are multi-modal medical images (MMI), the high requirement of spatial imagination capability for doctors still poses a challenge. Therefore, the multi-modal medical image fusion (MMIF) algorithm is the key to resolving this awkward situation [5]. Generally, MMIF is a process of combining salient and complementary information into images with high visual perceptual quality, thereby benefiting more comprehensive and accurate disease diagnosis and treatment.

Currently, MMIF methods are mainly divided into traditional and deep learning (DL) based fusion methods. The former consists of three parts: image decomposition and reconstruction, image fusion rules, and image quality assessment [6]. The traditional methods do not require model training but need to fix specific fusion strategies in advance. However, manually designed complex image decomposition methods are usually ineffective in retaining important information from the source images and may produce artifacts in the fused image. In addition, feature extraction methods are usually designed for specific tasks, leading to the poor generalization ability and robustness of the fusion methods.

As for existing DL-based [7] methods, they have improved the fusion quality to some extent, but their fusion effect is greatly influenced by the lack of gold standards, the limitations of the adopted network structure and improper loss function. Besides, unlike many traditional methods, previous DL-based fusion methods have rarely used feature decomposition. Recently, Zhao et al. [8] have proposed the Correlation-Driven Dual-Branch Feature Decomposition based fusion (CDDFuse) method, which combines Convolutional Neural Network (CNN) with Vision Transformer (ViT). In CDDFuse, the distinction between cross-modal features and shared features is facilitated to increase the correlation between the low-frequency features and decrease the correlation between the high-frequency features. However, when handling low-resolution MMI, rich detailed textures and blurred edges, CDDFuse cannot always work well. One example is that the CDDFuse does not perform well in CT-MR fusion in RIRE dataset [9] with large amounts of low-frequency monochromatic smearing, i.e., a pure grey background of the image covers the detailed textures of the MR image. Therefore, the following drawbacks can not be ignored. Firstly, the network uses a two-stage training strategy in which the cascading structure of image fusion and image reconstruction modules is trained in a serial way, leading to the accumulation of errors. Moreover, the feature decomposition network involves insufficient feature interaction, resulting in the deterioration of complementary information. Finally, the loss function used in CDDFuse cannot ensure the preservation of smooth boundary transition and high-quality visual fidelity.

To address the above-mentioned problems of the CDDFuse, we have proposed the Coupled Image Reconstruction and Fusion (CIRF) strategy. In this strategy, we have optimized the network structure and applied a new loss function. Our contributions can be briefly summarized as:

- We have proposed a novel fusion network with parallel image fusion and image reconstruction modules that share the same encoder and use the image masking strategy to enhance the feature learning ability of the encoder, thereby reducing error accumulation.
- The base-detail feature decomposition is optimized by adopting a concise parallel ViT-CNN structure, where base-details are processed separately but interact with each other to facilitate producing the complementary information, making the feature decomposition more effective.
- A new loss function combination is applied, i.e., the weighted sum of the reconstruction loss and the fusion loss. The former takes into account detail recovery, structural fidelity, and edge preservation. The latter utilizes a powerful unsupervised evaluation function.
- The performance of our method has been evaluated on three datasets with five types of multi-modal samples, and it demonstrates superior fusion performance to several traditional and DL-based fusion algorithms.

## 2. Related Works

### 2.1. The Traditional Fusion Methods

Image fusion has been extensively studied before the prevalence of DL. The traditional fusion methods have used relevant mathematical transformations to manually analyze the activity level and design fusion rules in the spatial or transform domain [10].

Spatial domain based fusion methods typically compute a weighted average of the local or pixel-level saliency of the two source images to obtain a fused image. However, these methods usually have problems in pseudo-color-image decomposition, i.e., the base and detail images obtained after decomposition are in grayscale. To tackle this problem, Du et al. [11] have come up with the Adaptive Two-scale Image Fusion (ATF) method, which uses Otsu’s method [12,13] to decompose the pseudo-color input image into a base image and a detailed image, thereby obtaining an adaptive threshold for two-scale image fusion [14].

Transform domain-based fusion methods usually start by transforming the source images into the transform domain (e.g., wavelet domain [15]) to obtain different frequency components. For instance, Yin et al. [16] have proposed a medical image fusion method in the Nonsubsampled Shearlet Transform (NSST) domain [17]. Firstly the high frequency bands and low frequency bands are obtained by performing NSST decomposition of the input image. Then, the high-frequency bands are fused by the PAPCNN model [18]. As for the low-frequency bands, two new measures of activity level are introduced, namely the Weighted Local Energy (WLE) and the Weighted Sum of Eight-neighborhood-based Modified Laplacian (WSEML). WLE is utilized to address the issue of energy loss that arises due to the average-based conventional low-frequency fusion rule, and WSEML is fully employed to extract the detailed information present in the low-frequency band. The fused high-frequency and low-frequency bands are passed through inverse NSST to generate the final fused image. Besides, Li et al. [19] have proposed the Laplacian Redecomposition (LRD) framework. Here, the source images are processed by Gradient-domain Image Enhancement (GDIE) which is used for increasing the LRD ability of detail extraction through mapping gradient information adaptively. Then, the enhanced image undergoes Laplacian pyramid (LP) transform [20] to decompose it into the High-frequency Subband Image (HSI) with edges and details and the Low-frequency Subband Image (LSI) with background information. Through the pre-set fusion rules, image fusion is performed both on HSI and LSI to generate the high and low-frequency components of the fused image, respectively. Eventually, these components are subjected to inverse LP to produce the final fused image.

### 2.2. The DL-Based Fusion Methods

At present, the two most commonly used models in image fusion are CNN and Transformer. However, due to the giant computational overhead, pure Transformer methods are rare and CNN-Transformer hybrid networks are often used for image fusion.

#### 2.2.1. The CNN Based Image Fusion

The most popular DL network in image processing is CNN. By training a CNN model, it is capable of recognizing and extracting different features for image fusion. Usually, in a CNN with multiple layers, each network layer produces several feature maps which are calculated through convolution, spatial pooling, and non-linear activation [21]. Besides, the CNN network can model the local area quite well by selecting an appropriate window size. However, it needs to stack very deep CNN layers to meet the requirement of the global perspective.

Some fusion methods usually contain CNN layers to extract multi-scale information. For example, Zhang et al. [22] have come up with a general image fusion framework based on a convolutional neural network (IFCNN). The most remarkable characteristic of this model is that it is fully convolutional so that it can be trained in an end-to-end manner without any post-processing procedures. To avoid the loss of fusion capabilities when training a single model for different scenes sequentially, Xu et al. [23] have presented a unified unsupervised image fusion network, termed U2Fusion, to solve multiple-territory fusion problems. In addition, some fusion methods which are initially proposed for infrared-visible image fusion are also inspiring for MMIF. For example, Li and Wu have proposed a DL architecture named DenseFuse [24] which consists of an encoder, a fusion layer, and a decoder. To extract salient features from source images effectively, the encoder is constructed with convolutional layers and dense blocks where the output of each layer is used as the input of all the subsequent layers. This prevents excessive information loss within the encoder. Li et al. have introduced an image fusion architecture, i.e., NestFuse [25], by developing a nest connection network and spatial/channel attention models. To begin with, they use pooling-assisted convolution to extract the multi-scale features. Then, several proposed spatial/channel attention models are utilized to fuse these multi-scale deep features in each scale. Li et al. [26] have also proposed a residual fusion network (RFN) based on a residual architecture to replace the traditional fusion approach. The learning of model parameters is accomplished by a novel two-stage training strategy. In the first stage, an auto-encoder network based on Nest connection is trained for better feature extraction and image reconstruction ability. Next, the RFN is trained using a specially designed loss function for fusion.

#### 2.2.2. The CNN-Transformer-Based Image Fusion

Another widely used paradigm is the Transformer [27]. As an architecture initially proposed for natural language processing (NLP), the Transformer works by using stacked layers of self-attention and feed-forward networks to deal with data sequences. In the field of computer vision (CV), the Vision Transformer (ViT) [28] has been proposed to extend the application of the attention mechanism. Its basic principle is to treat images as sequence data and use self-attention mechanisms to capture their spatial and temporal information. Firstly, the input images are divided into multiple patches (e.g., with the size of 16 × 16), flattened and concatenated with positional encoding, and projected into the Transformer encoder. Then, by calculating the correlation between embedded patches, attention weight distribution is obtained to enable the model to focus on different positions in the image, thereby facilitating better global information transmission.

Although the cascaded self-attention modules can capture global representations, the ViT still cannot perform well in extracting the positional-encoded features with low computational consumption. Hence, the idea of using the convolution operators to extract local features and the self-attention mechanisms to capture global representations has been presented. For the MMIF, Tang et al. [29] have proposed an adaptive Transformer to capture long-range dependencies, which improves the global semantic extraction ability. They also make use of adaptive convolution instead of vanilla convolution to modulate the convolutional kernel automatically based on the wide-field context information. Zhang et al. [30] have introduced the Transformer as the fusion block, and applied multi-scale CNN as encoders and decoders. By interacting across fusion Transformers at multiple scales, the global contextual information from different modalities is incorporated more effectively. Zhou et al. [31] have proposed a novel architecture that combines a densely connected high-resolution network (DHRNet) with a hybrid transformer. Specifically, the hybrid transformer employs the fine-grained attention module to generate global features by exploring long-range dependencies, while the DHRNet is responsible for local information processing. Liu et al. [32] have used a CNN and Transformer module to build the extraction network and the decoder network. Besides, they have designed a self-adaptive weighted rule for image fusion.

## 3. Proposed Method

In this section, we present the architecture of CIRF and explain how each component works. Then, we introduce the entire model workflow and the loss function.

### 3.1. Framework of CIRF

Our CIRF consists of two parallel branches. The fusion branch adopts an encoder-decoder architecture with feature decomposition, fusing the base and detail features separately. The reconstruction branch, as a multi-task branch, assists in training a more powerful encoder and contributes to the reduction in the overall loss. The two branches share one common encoder so that ViT and CNN are parallel while the subsequent branch modules are different, and they complete the reconstruction and fusion tasks, respectively. In each epoch, the weighted summation of the reconstruction loss and the fusion loss is performed.

**Figure 1:**
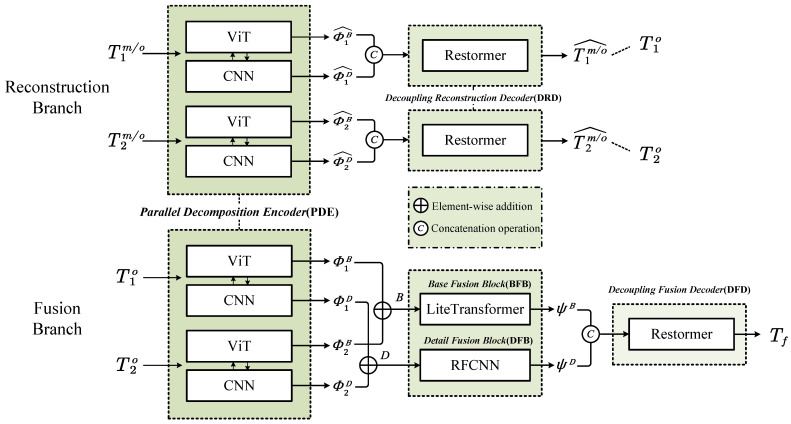
The brief workflow of the CIRF network. The architecture consists of two branches: the reconstruction branch at the top and the fusion branch at the bottom. During training, both branches are calculated simultaneously, and their total loss is added by an adjustable weight. However, in model inference, only the fusion branch is retained.

As shown in Figure 1, the framework of CIRF contains a Parallel Decomposition Encoder (PDE), Decoupling Reconstruction Decoder (DRD), Base Fusion Block (BFB), Detail Fusion Block (DFB), and Decoupling Fusion Decoder (DFD). In the following, these modules will be referred to by abbreviation for simplicity and clarity.

Furthermore, to make narration easier, here we agree on some symbols.

- We use *o* and *m* to distinguish original and masked images, e.g., $T_{1}^{o}$ and $T_{1}^{m}$.
- We use $\hat{\left(\cdot \right)}$ to denote information extracted from masked inputs in the reconstruction branch, e.g., $\hat{\Phi_{1}^{B}}$ and $\hat{T_{1}^{m}}$.
- We use *B* and *D* to abbreviate base and detail, *r* and *f* to abbreviate reconstruction and fusion, e.g., $\phi^{B}$ and $\psi^{D}$.
- The outputs of the encoder, two fusion blocks, and two decoders are represented by $E\left(\cdot \right)$, $F_{b}\left(\cdot \right)$, $F_{d}\left(\cdot \right)$, $D_{r}\left(\cdot \right)$ and $D_{f}\left(\cdot \right)$, respectively.

### 3.2. Fusion Branch

#### 3.2.1. Overview

The fusion branch utilizes an encoder-fusion-decoder structure that involves feature decomposition. It has four components: PDE, BFB, DFB and DFD.

The inputs of this branch are two batches of original multi-modal images $T_{1}^{o}$ and $T_{2}^{o}$. These images are firstly decomposed into base and detail features through PDE, i.e., a paralleled ViT-CNN encoder, formulated as:

(1) $$\Phi_{1}^{B},\Phi_{1}^{D}=E\left(T_{1}^{o}\right)$$

(2) $$\Phi_{2}^{B},\Phi_{2}^{D}=E\left(T_{2}^{o}\right)$$

Then, two types of features are added for high-frequency and low-frequency information fusion, respectively. For BFB, a Lite Transformer (LT) [33] module with long-short-range attention is chosen. In essence, it is a Transformer that is assisted with the Gated Linear Unit (GLU) and convolution block, and thus it is suitable for long-range information fusion while taking into account the local details. For DFB, we have constructed the Residual Fusion CNN (RFCNN) which is a pure convolutional neural network with various residuals so as to keep more detailed information. This process can be expressed as:

(3) $$\psi^{B}=F_{b}\left(\Phi_{1}^{B}+\Phi_{2}^{B}\right)$$

(4) $$\psi^{D}=F_{d}\left(\Phi_{1}^{D}+\Phi_{2}^{D}\right)$$

Finally, the outputs of fusion blocks are concatenated and sent into DFD (Restormer module) [34] for image restoration until we obtain:

(5) $$T_{f}=D_{f}\left(cat\left\{\psi^{B},\psi^{D}\right\}\right)$$

#### 3.2.2. Parallel Decomposition Encoder

**Figure 2:**
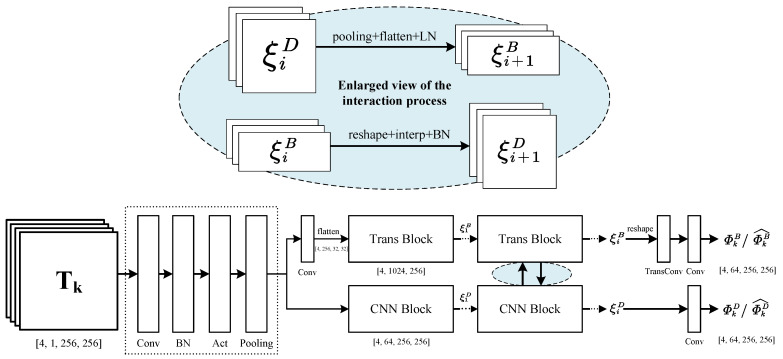
The brief workflow of PDE. The overall architecture comprises two parallel branches for feature decomposition, the ViT branch and the CNN branch, which are also connected with each other to form information complementation. Here, the inputs are two modalities of single-channel gray-scale images denoted as $T_{k}$, where k=1,2. In the experiments, we fix N=4, H=W=256, C=1. Therefore, after data pre-processing, the input tensor dimension can be written as [4,1,256,256] while the output tensor will be [4,64,256,256]. The complementary process is illustrated by feature map transformations shown at the top of the figure.

When it comes to traditional multi-modal medical image fusion (MMIF) methods, there have been several strategies based on frequency decomposition, but most of them are ineffective and time-consuming. In CDDFuse [8], a dual-branch Transformer-CNN framework that performs cross-modal feature decomposition extraction through a shared encoder is proposed and has obtained relatively good results. However, in the specific scene of MMIF, given the low-resolution input images, the detail loss caused by CDDFuse is more serious, thereby leading to contrast distortion and obvious artifacts. Inspired by [35,36], we have developed a lite encoder that can retain detail representations and base features to the maximum extent, whose framework is shown in Figure 2.

Here, the inputs of the network can be denoted as a four-dimensional matrix $\left[N,C,H,W\right]$, which represents the batch size, channel, height and width, respectively. Generally, most medical images are single-channel gray-scale images. While processing an RGB image, we first convert it into YUV space, where the Y channel contains gray-scale information, and then fuse the Y channel with another gray-scale image separately. Finally, we re-stitch the image with UV channels to restore a colored one [16]. Accordingly, after data pre-processing, the input tensor can be unified as $\left[N,1,H,W\right]$.

In PDE, the input tensor is initially processed by a coarse feature extraction module with large convolution kernels (e.g., 7 × 7) and pooling layers, and then it is sent to two parallel branches comprising multi-head Transformer Block and CNN Block. Notably, features input into the Transformer Block need to go through an extra convolution layer before being reshaped into 8 × 8 patches [28]. By doing so, the number of feature channels is increased and the size of the feature maps is reduced, which is more conducive to effective and efficient feature extraction by attention layers. The number of heads in the self-attention layer is set to 4, and the stack depth is set to 6 with a drop rate of 10%.

Subsequently, the ViT and CNN Blocks are repeatedly stacked for *i* times. Considering the complementation of base and detail features [35], we have added information interaction between the multi-head Transformer and CNN Block when i⩾2, which contributes to better preserving detailed texture features and protecting image edge contours. During the transformation from detail feature maps (e.g., $\xi_{i}^{D}$) to base ones (e.g., $\xi_{i+1}^{B}$), pooling, flattening, and layer-normalization operations are applied. On the contrary, reshaping, interpolation, and batch-normalization operations are adopted for transforming from base feature maps to detail ones (e.g., from $\xi_{i}^{B}$ to $\xi_{i+1}^{D}$). Experiments show that setting i=2 is enough to obtain satisfactory outcomes and can help to limit network parameters to a relatively small scale.

Eventually, through reshaping and trans-convolution, we can restore the feature maps back to their original visible sizes. However, the extracted deep-layer information has increased, which can be described as $\left[N,64,H,W\right]$.

#### 3.2.3. Base and Detail Fusion Block

For the MMI, it is still important to pay close attention to the local features when fusing the global information in BFB. Unfortunately, the traditional Transformer architecture can be inefficient due to its large time and space consumption as well as computational redundancy. To tackle this, a CNN-assisted lite Transformer which offers a trade-off between the feed-forward computation for wider attention layers is applied [33]. Here, one group of heads is responsible for the local context modeling via convolution, while the other conducts long-distance relationship modeling via attention.

**Figure 3:**
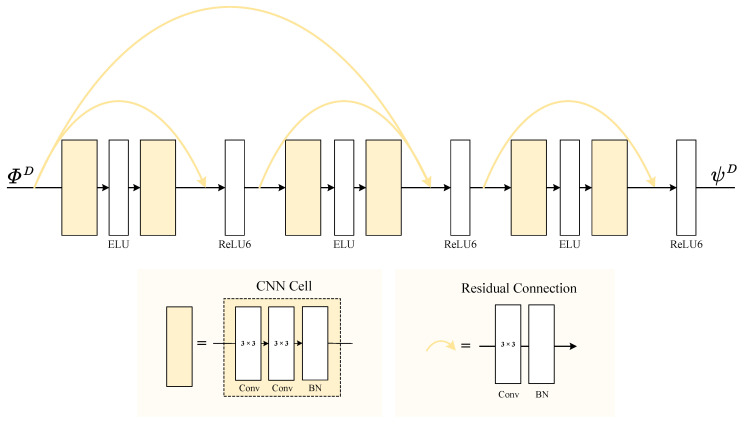
The architecture of RFCNN. In this figure, the yellow box and the yellow line respectively represent different CNN modules with a small kernel size of 3 × 3. Notably, the ELU and ReLU6 activation functions are specifically used to enhance expressivity and prevent gradient explosion. By adding residuals, RFCNN can effectively accomplish detail-feature fusion tasks from $\Phi^{D}$ to $\psi^{D}$.

As for DFB, reducing information loss is the most urgent goal. Therefore, we should not only improve the richness of information (i.e., improve the dynamic range of output representation) but also prevent gradient explosion and model non-convergence. As shown in Figure 3, a simple CNN cell (the yellow box) and a residual line (the yellow line) composed of convolution layers and batch normalization are first defined. Additionally, between two CNN cells comes an Exponential Linear Unit (ELU) [37] activation function, which is unilaterally saturated and outputs tensors with zero-mean distribution, thereby speeding up training and accelerating convergence. Besides, we have utilized convolutional residuals to link the output of the front module to the input of the rear module with a ReLU6 activation function [38] added after post-merger residuals. By doing so, the output is limited to the maximum of 6, thereby preventing gradient explosion, benefiting gradient descent at low precision, and improving decimal expression ability [39]. Under such an architecture of detail feature fusion, detail fidelity will be ensured by continuous optimization.

#### 3.2.4. Decoupling Fusion Decoder

To restore noise-disturbed images, Zamir et al. have developed an efficient Transformer model [34] that can output high-resolution images in restoration tasks. This is also used in [8] for fused image decoding. In this paper, we retain this module.

### 3.3. Reconstruction Branch

In RFN-Nest [26], a two-stage training strategy has been presented for the first time. By pre-training the network via reconstruction tasks, the quality of the fused image is greatly improved, which also alleviates the challenge caused by the lack of gold standard to some extent. However, two-stage training can cause error accumulation, raising a stage time allocation problem, and resulting in redundant time overhead and low robustness. Therefore, a multi-task network that couples the reconstruction branch and the fusion branch with one common encoder is proposed. Here, the reconstruction branch aims at training a more powerful feature extraction encoder. By paralleling the two stages, the total loss of the task can better reflect model capability at any time.

Besides, inspired by [40], we have figured out that in some cases (e.g., when given low-quality source images), adding random image masks can enhance the expressivity of the shared encoder. Hence, the encoding process can be characterized by:

(6) $$\hat{\Phi_{1}^{B}},\hat{\Phi_{1}^{D}}=E\left(T_{1}^{m/o}\right)$$

(7) $$\hat{\Phi_{2}^{B}},\hat{\Phi_{2}^{D}}=E\left(T_{2}^{m/o}\right)$$

Then, features derived from the same image are concatenated and fed into the shared PDE that will be discarded later. Since the reconstruction branch mainly contributes to the encoder, the Restormer module used in Section 3.2.4 is again selected here as DRD for convenience. Here, it can be any simple decoding structure. The function of DRD can be expressed as:

(8) $$\hat{T_{1}^{m/o}}=D_{r}\left(cat\left\{\hat{\Phi_{1}^{B}},\hat{\Phi_{1}^{D}}\right\}\right)$$

(9) $$\hat{T_{2}^{m/o}}=D_{r}\left(cat\left\{\hat{\Phi_{2}^{B}},\hat{\Phi_{2}^{D}}\right\}\right)$$

Here, it is worth mentioning that in the inference process, the reconstruction branch will be cut off.

### 3.4. Loss Function

The workflow of the reconstruction branch is a supervised process with a given ground truth (i.e., source images). Therefore, the reconstruction loss is composed of three components: mean square error (MSE), structural similarity (SSIM) [41] and spatial gradient loss (SG) [42,43]. For each source image *k*, the reconstruction loss $L_{rec,k}$ can be calculated by:

(10) $$L_{rec,k}=L_{MSE}\left(\hat{T_{k}^{m/o}},T_{k}^{o}\right)+\alpha \cdot L_{SSIM}\left(\hat{T_{k}^{m/o}},T_{k}^{o}\right)+\beta \cdot L_{SG}\left(\hat{T_{k}^{m/o}},T_{k}^{o}\right),k=1,2.$$

where α, β are adjustable weights; $L_{MSE},\;L_{SSIM},\;L_{SG}$ protect the local pixel information, regional structure information, and edge contour information, respectively. Meanwhile, the gradient loss can be described as $L_{SG}=\frac{1}{HW}\sum |||\nabla \hat{T_{k}^{m/o}}|-|\nabla T_{k}^{o}{\left||\right|}_{1}$.

Furthermore, the total reconstruction loss is computed as:

(11) $$Loss1=\mu \cdot \left(L_{rec,1}+L_{rec,2}\right)$$

where μ is a weight for numeral balance, i.e., adjusting the order of magnitude.

On the other hand, the fusion branch lacks ground truth, so the unsupervised loss function should be able to effectively measure the intensity correlation and structural information between the source and fused images. Inspired by [39], we choose mutual information (MI), the sum of the correlations of differences (SCD) [44], structural similarity (SSIM), and edge retentiveness ($Q^{\mathrm{AB}/F}$) [45] as our four metrics that make up the fusion loss function. Such a function can be described as:

(12) $$Loss2=\lambda \cdot \left(L_{MI}+L_{SCD}\right)+\left(1-\lambda \right)\cdot \left(L_{SSIM}+L_{Q^{AB/F}}\right)$$

where λ is a hyper-parameter and $L_{MI},\;L_{SCD},\;L_{SSIM},\;L_{Q^{AB/F}}$ reflect the amount of common information, the correlations of image differences, the similarity of luminance, contrast and structure as well as the preservation of edge information, respectively. For each metric in Equation (12), each loss is one minus the normalized average of each metric of the two source images and the fused image.

As a whole, the total loss function is as follows:

(13) $$Loss_{total}=\left(1-\sigma \right)\cdot Loss1+\sigma \cdot Loss2$$

where σ is also a hyper-parameter to balance our network’s preference for reconstruction and fusion, which will be discussed later in the ablation study.

## 4. Experimental Settings

In this section, we discuss the settings of our dataset, the compared algorithms and the metrics we have chosen to evaluate the algorithms.

### 4.1. Dataset

The Whole Brain Atlas (Atlas) [46], the IXI Brain Development Dataset (IXI) [47], and the Retrospective Image Registration Evaluation (RIRE) [9] are used to evaluate our algorithm. The Atlas dataset collected by Harvard Medical School includes CT, MR, PET, and SPECT images from patients with various diseases. The IXI dataset collected by three hospitals in London includes 3D MR images from 600 healthy test-takers, including T1-, T2-, and PD-weighted images. The RIRE dataset collected by the National Institute of Biomedical Imaging and Bioengineering includes CT, MR, and PET images.

**Figure 4:**
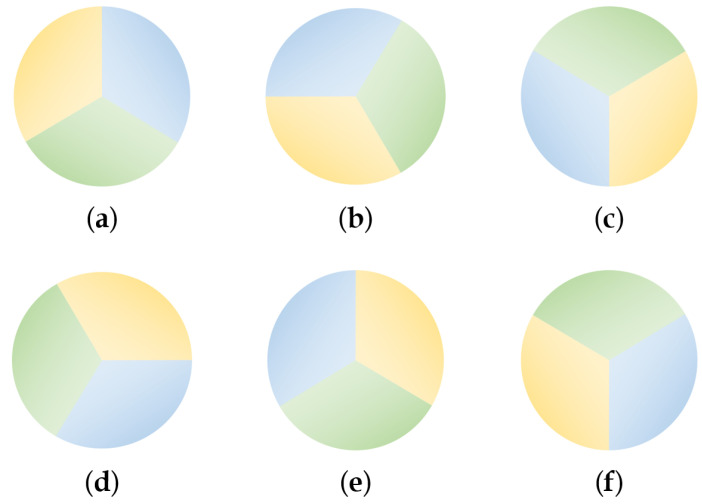
Visualization of training dataset flipping via a three-color-leaf example. (**a**) Original image; (**b**) $90^{\circ }$ counterclockwise rotation; (**c**) $180^{\circ }$ counterclockwise rotation; (**d**) $90^{\circ }$ clockwise rotation; (**e**) Left-right mirror symmetry; (**f**) Up-down mirror symmetry.

In the Atlas dataset, we have acquired three groups of images, including 388 pairs of SPECT-CT/MR images, 590 pairs of multi-modal MR (i.e., T1-T2) images, and 140 pairs of CT-MR images. For multi-modal MR image pairs, we have randomly divided them into training and testing sets in the ratio of 8:1. To increase the number of training images, we have augmented the training set by performing rotations and mirroring operations on the original images, resulting in six times the amount of data. The generated methods are shown in Figure 4. SPECT-CT/MR and CT-MR images in the Atlas dataset are all retained to test images for evaluating model generalization ability.

In the IXI and RIRE datasets, we have, respectively, acquired 3936 multi-modal MR (i.e., PD-T2) image pairs and 476 CT-MR image pairs. We have used similar methods to process the dataset, i.e., dividing them into training and testing sets in a ratio of 8:1. Considering that the IXI images are enough for training and testing, we have only enhanced the training set of the RIRE dataset. It is noteworthy that we have first registered the RIRE dataset using the Elastix algorithm [48,49], and then used these registered image pairs to produce training and testing sets. Specifically, the MR images and CT images are chosen as the fixed and moving images, respectively, for registration. See Table 1.

### 4.2. The Comparison Algorithms

Our method will be compared with several traditional algorithms (i.e., PAPCNN [16], TIF [11], and ReLP [19]) and DL-based algorithms (i.e., CDDFuse [8], DenseFuse [24], IFCNN [22], NestFuse [25], U2Fusion [23], and RFN-Nest [26]). For the traditional methods, the fusion results are directly obtained by running the corresponding algorithm. For DL-based methods, we have trained the U2Fusion on the datasets mentioned above. However, for other methods, we have directly used the models trained by their corresponding authors as these models are already sufficiently trained.

### 4.3. Fusion Metrics

We have used eight metrics to evaluate our algorithm. Standard deviation (SD) measures the contrast of the fused image. Peak-signal-to-noise ratio (PSNR) measures the effective signal intensity of the fused image. For the computation of PSNR, the two mean square errors (MSE) between the source images and the fused image are first averaged to produce the mean MSE. Then, the ratio of the square of the maximum pixel intensity to the mean MSE is computed, and the logarithm (base 10) of the ratio is multiplied by 10 to produce the PSNR according to [50]. The sum of the correlations of differences (SCD) measures distortion and loss of information of the fused image [44]. Mutual information (MI) measures the amount of information from the original images that is captured in the fused image. The structural similarity (SSIM) evaluates the structural similarity between the fused image and the source image, the overall SSIM is calculated by directly averaging the two SSIM values of the two source images and the fused image according to [51]. $Q^{\mathrm{AB}/F}$ evaluates the edge information from the original image [45]. The visual information fidelity for fusion (VIFF) evaluates the quality of an image based on the calculation of visual information fidelity [52]. The ratio of spatial frequency error (|rSFe|) evaluates the ratio of spatial frequency error calculated from the source image referred to as SF. A value of |rSFe| greater than zero indicates the introduction of noise during image fusion, while a value less than zero indicates the loss of information [53]. In general, the closer the |rSFe| is to 0, the better the fusion effect. However, larger values of other metrics indicate better fusion performance.

## 5. Ablation Experiments

Our algorithm is realized using Python 3.10, Pytorch 2.0.1 on Ubuntu 22.04.3 LTS, and CUDA 11.8. Meanwhile, it is run on a server with the Intel(R) Xeon(R) Gold 6248R CPU (Intel, Santa Clara, CA, USA) and the NVIDIA RTX A100 with 40 G VRAM (NVIDIA, Santa Clara, CA, USA). Additionally, we use the Adam optimizer to update the model parameters.

In the DL-based MMIF tasks, the loss function is extremely important. Here, our loss function has two adjustable hyper-parameters, λ (in Equation (12)) and σ (in Equation (13)) which will be determined subsequently. Moreover, we have also explored the impact of inputting images with different masking ratios based on the considerations that in some cases masking can enhance the feature extraction capability of PDE and reduce the fusion artifacts. To obtain the optimal values for the above three parameters, we have conducted ablation experiments on three datasets separately.

### 5.1. Parameter Setting on Atlas Dataset

On the Atlas dataset, we have firstly fixed the values of parameters λ and σ based on our experience, and then found the best value for the masking ratio by increasing it with a step size of 0.1. The results are shown in Table 2. Obviously, the value of MI reaches the maximum when the masking ratio is 0.1, while the values of SCD and VIFF decrease with the increase in the masking ratio. Besides, by setting the masking ratio to 0.1, we observe fewer fusion artifacts in the fused results compared with those produced with the masking ratio = 0. Taking all these into account, we will set the masking ratio = 0.

Next, to make the fusion branch achieve the best effect, we have fixed the masking ratio at 0.1 and preset σ at 0.2 while altering the value of λ. According to the metric values in Table 3, we will choose λ=0.3 to achieve the trade-off among all evaluation indicators.

Furthermore, to balance the performance of the two model branches, another parameter σ needs to be determined. Thus, we have fixed the masking ratio and λ at their optimal value (i.e., masking ratio = 0.1 and λ=0.3), and changed the value of σ. As shown in Table 4, when σ is set to 0.2, relatively high SCD, MI, SSIM, $Q^{AB/F}$ and VIFF values can be obtained, so we choose σ=0.2. Additionally, the comparison of the metrics when σ=1.0 and σ=0.2 clearly demonstrates the contribution of the reconstruction branch to the PDE.

### 5.2. Parameter Setting on IXI Dataset

To ensure the rigor of the experiment and verify the robustness of our method, we have further used two other datasets to compute the metric value with different masking ratios, λ and σ. The results from using two different masking ratios are shown in Table 5. Due to the high quality of source images of the IXI dataset (i.e., rich and clear details, few artifacts), adding masking to the original images will not improve the fusion effect. Therefore, we set the masking ratio to 0 to obtain the optimal results.

From the results using different λ in Table 6, we can see that as λ increases, PSNR generally improves firstly but then experiences a decline. Moreover, with increasing λ, SD, SCD and VIFF also increase while $Q^{AB/F}$ decreases. Based on the above analysis, we will set λ=0.3.

From the results using different σ in Table 7, we can see that all metrics have no obvious changes but minor fluctuations. However, as σ increases, PSNR, MI, SSIM and $Q^{AB/F}$ reach their maximum when σ=0.2 while other metrics are also competitive. Accordingly, we will fix σ=0.2.

### 5.3. Parameter Setting on RIRE Dataset

As for the RIRE dataset, we have computed the metrics using different masking ratios. Table 8 indicates that by inputting non-masked source images, our method works best.

From the results of λ in Table 9, it can be seen that the value of SD reaches the maximum and the value of |rSFe| reaches the minimum when λ is equal to 0.3. Meanwhile, the values of PSNR, MI, and $Q^{AB/F}$ are also relatively high with λ=0.3. Based on comprehensive consideration, we fix λ=0.3.

The results from using different σ in Table 10 show that when σ equals 0.4, SSIM reaches the maximum while SCD, MI, $Q^{AB/F}$ and VIFF achieve relatively high values. Therefore, we choose σ=0.4.

After testing on three datasets, we have found that our algorithm performs best when the parameter λ is set to 0.3. However, the optimal values of masking ratio and σ vary with the characteristics of different images.

## 6. Qualitative Analysis

### 6.1. The Results of the Atlas Dataset

For the CT-MR and SPECT-CT/MR image pairs from the Atlas dataset, we have not trained a specific CIRF on them but directly used the model trained on T1-T2 image pairs.

**Figure 5:**
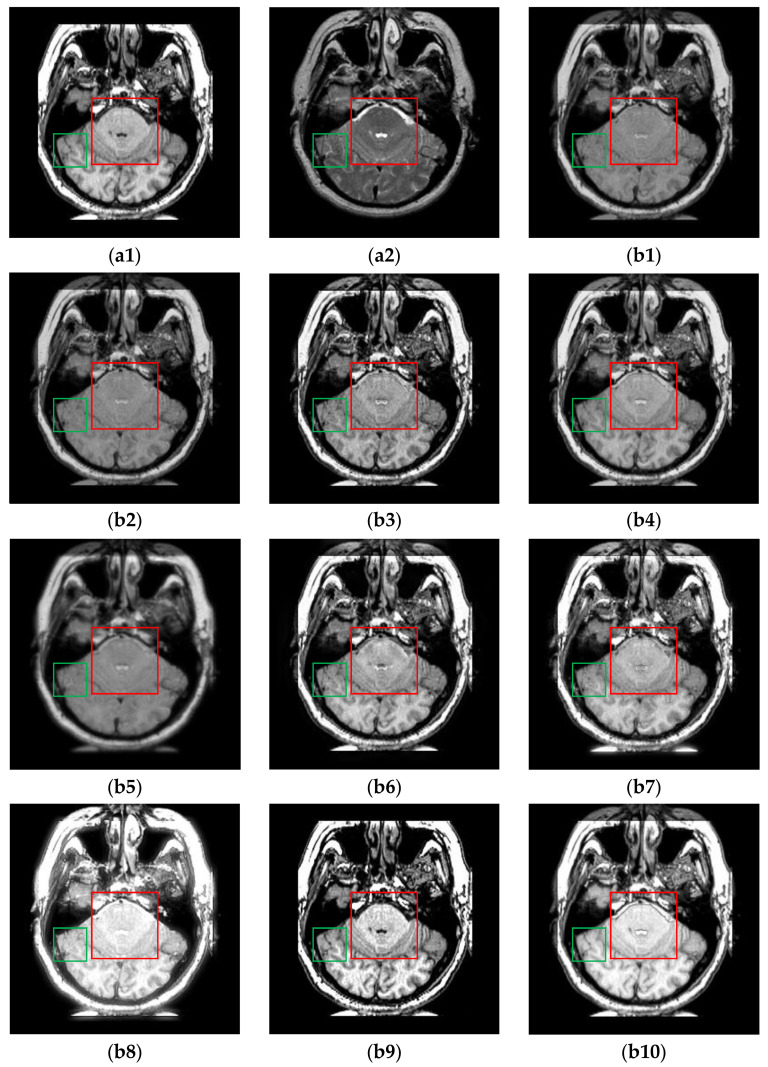
The results of all algorithms on the multi-modal image pairs from Atlas dataset. (**a1**) Source T1 image; (**a2**) Source T2 image; (**b1**) U2Fusion; (**b2**) DenseFuse; (**b3**) IFCNN; (**b4**) NestFuse; (**b5**) RFN-Nest; (**b6**) PAPCNN; (**b7**) ReLP; (**b8**) TIF; (**b9**) CDDFuse; (**b10**) CIRF.

The fusion results of multi-modal MR (i.e., T1 and T2) image pairs from the Atlas dataset are shown in Figure 5. Generally, except for TIF, CDDFuse and CIRF, the brightness and intensity of all other algorithms are insufficient. Specifically, from the areas marked by the red boxes, the upper parts of the brainstem are blurred or missing in the fused results of U2Fusion, DenseFuse, RFN-Nest, PAPCNN, ReLP and CDDFuse. Additionally, as labeled by the green boxes, except for CDDFuse and CIRF, all other methods produce blurry and incomplete boundaries of the occipital lobe. By comparison, the CIRF algorithm performs better than other algorithms in terms of edge preservation.

**Figure 6:**
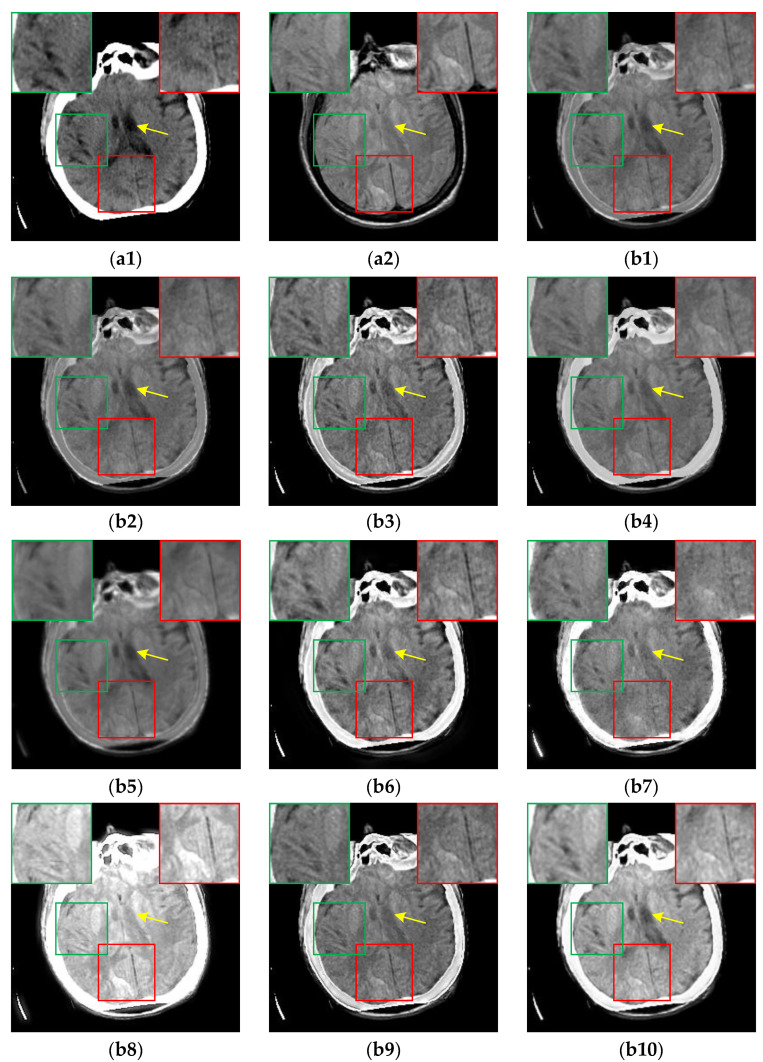
The results of all algorithms on the CT-MR image pairs from Atlas dataset. (**a1**) Source CT image; (**a2**) Source MR image; (**b1**) U2Fusion; (**b2**) DenseFuse; (**b3**) IFCNN; (**b4**) NestFuse; (**b5**) RFN-Nest; (**b6**) PAPCNN; (**b7**) ReLP; (**b8**) TIF; (**b9**) CDDFuse; (**b10**) CIRF.

The fusion results of the CT-MR image pairs are shown in Figure 6. Clearly, ReLP, TIF and CIRF outperform other methods in preserving the white cranium cross-section from the CT image. From the green boxes and the yellow arrows, we can clearly observe that only U2Fusion, DenseFuse and CIRF can simultaneously preserve low-intensity information and retain crucial information from the CT image. However, CIRF produces the fused result with higher contrast than U2Fusion and DenseFuse. The fused results marked with the red boxes show that CIRF can preserve the details from MR images better than other methods. Therefore, it is evident that CIRF simultaneously retains the features derived from both CT and MR images, which indicates its strong feature extraction and fusion capability.

**Figure 7:**
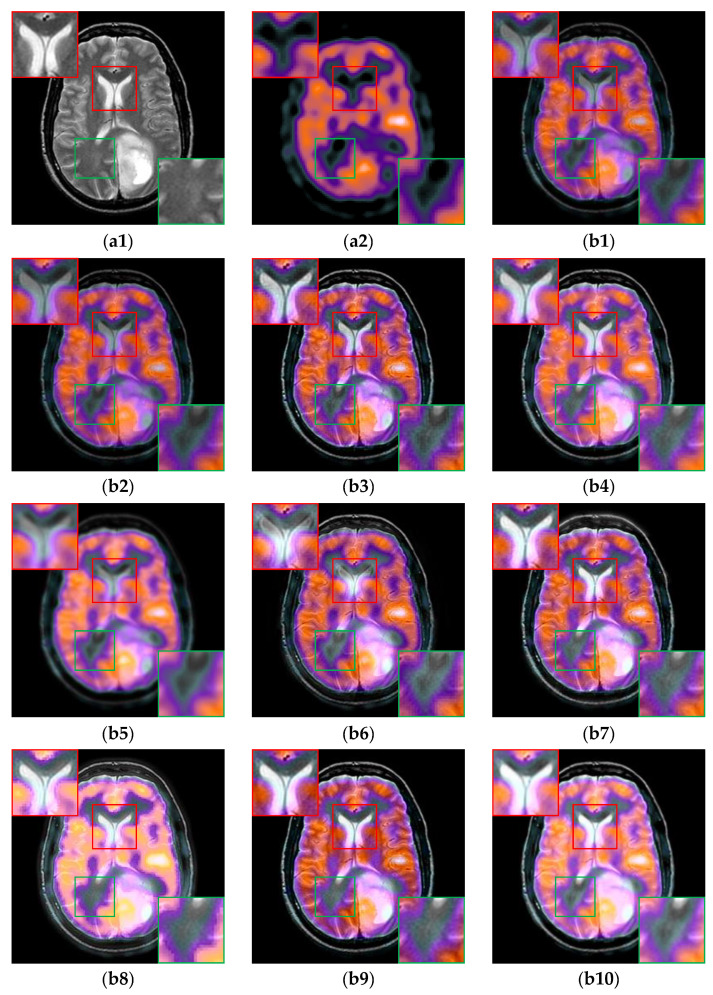
The results of all algorithms on the SPECT-CT/MR image pairs from Atlas dataset. (**a1**) Source CT/MR image; (**a2**) Source SPECT image; (**b1**) U2Fusion; (**b2**) DenseFuse; (**b3**) IFCNN; (**b4**) NestFuse; (**b5**) RFN-Nest; (**b6**) PAPCNN; (**b7**) ReLP; (**b8**) TIF; (**b9**) CDDFuse; (**b10**) CIRF.

The fusion results of SPECT-CT/MR image pairs are shown in Figure 7. As indicated by the red boxes, U2Fusion, DenseFuse and RFN-Nest cannot maintain the sharpness of details from MR image, and IFCNN, PAPCNN and TIF produce the unwanted ringing artifacts. As shown by the green boxes, NestFuse, ReLP and CDDFuse reduce the contrast of the details from the SPECT image. By comparison, CIRF can not only avoid undesirable artifacts but also preserve the important details from MR and SPECT images effectively. These results also indicate that CIRF has a good generalization ability when applied to different datasets.

### 6.2. The Results of the IXI Dataset

**Figure 8:**
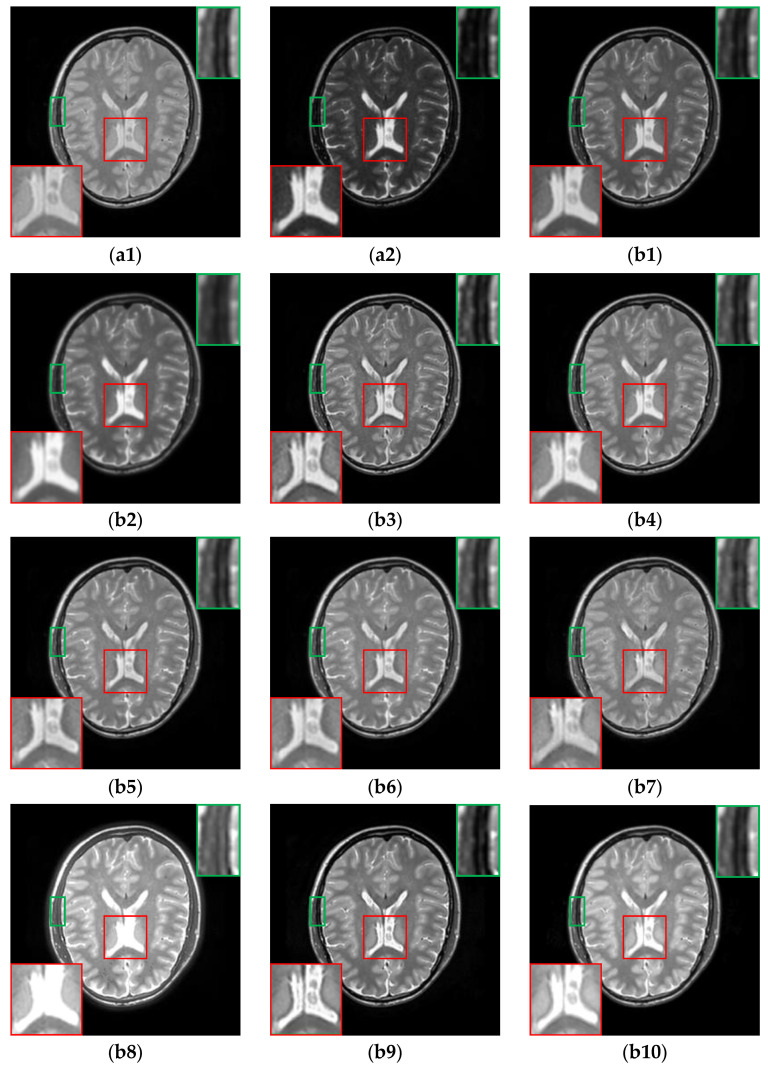
The results of all algorithms on the multi-modal MR image pairs from IXI dataset. (**a1**) Source PD image; (**a2**) Source T2 image; (**b1**) U2Fusion; (**b2**) DenseFuse; (**b3**) IFCNN; (**b4**) NestFuse; (**b5**) RFN-Nest; (**b6**) PAPCNN; (**b7**) ReLP; (**b8**) TIF; (**b9**) CDDFuse; (**b10**) CIRF.

For the multi-modal MR image pairs in the IXI dataset, the results are shown in Figure 8. From the fusion results marked by the red boxes, we can see that TIF damages the low-intensity information seriously, and ReLP causes the loss of some low gray-scale detail information in that the contour of the ventricular boundary is missing in Figure 8(b7). Compared with U2Fusion, DenseFuse and CDDFuse, CIRF maintains the continuity of the gray line in the green box, which indicates that CIRF can preserve the fine edges better.

### 6.3. The Results of the RIRE Dataset

**Figure 9:**
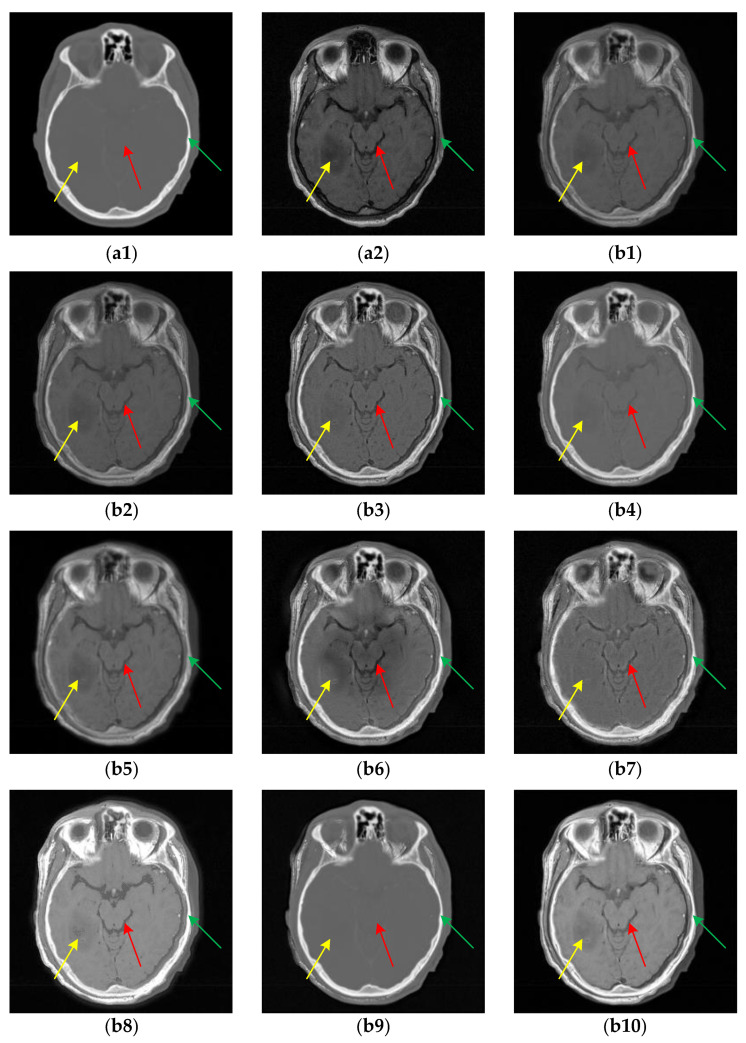
The results of all algorithms on the CT-MR image pairs from RIRE dataset. (**a1**) Source CT image; (**a2**) Source MR image; (**b1**) U2Fusion; (**b2**) DenseFuse; (**b3**) IFCNN; (**b4**) NestFuse; (**b5**) RFN-Nest; (**b6**) PAPCNN; (**b7**) ReLP; (**b8**) TIF; (**b9**) CDDFuse; (**b10**) CIRF.

For the CT-MR image pairs from the RIRE dataset, the results are shown in Figure 9. It is evident that CIRF produces clearer image details, higher image contrast, and less loss of original information. However, U2Fusion, DenseFuse and RFN-Nest fail to effectively fuse the bright cranium from the CT image as depicted by the green arrows. As shown by the yellow arrows, IFCNN, NestFuse, PAPCNN, ReLP and CDDFuse fail to retain the low-intensity areas in the MR image, and TIF produces blocky artifacts. Besides, NestFuse and CDDFuse result in a serious loss of structural information as pointed out by the red arrows.

## 7. Quantitative Evaluation

To quantitatively evaluate the fusion performance datasets, we have computed eight metrics of ten algorithms on three datasets. Table 11 lists the mean and deviation of each algorithm’s values across all datasets, where the deviation refers to the dispersion of all values of each metric from their mean [39].

As can be seen from Table 11, CIRF has significant advantages over all other algorithms in terms of SD, PSNR, VIFF and SCD. Meanwhile, CIRF can provide relatively close $Q^{AB/F}$ and |rSFe| to CDDFuse.

**Figure 10:**
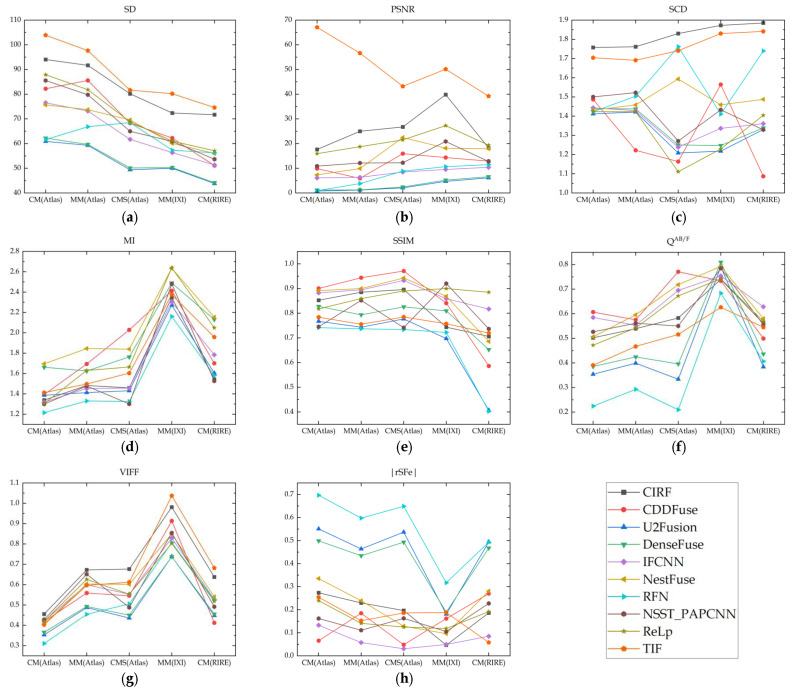
The results of eight metrics of all computed algorithms (**a**) SD; (**b**) PSNR; (**c**) SCD; (**d**) MI; (**e**) SSIM; (**f**) $Q^{AB/F}$; (**g**) VIFF; (**h**) |rSFe|.

Figure 10 shows the values of eight metrics for various algorithms on five kinds of multi-modal image pairs in three datasets. Overall, CIRF outperforms all other algorithms in terms of SCD and its VIFF achieves the highest value across the datasets except for IXI and RIRE. In addition, CIRF is only outperformed by TIF in PSNR and SD. Furthermore, CIRF achieves the most competitive |rSFe| on the IXI dataset, and provides comparable |rSFe| to CDDFuse on other datasets.

## 8. Conclusions

This paper has come up with a coupled reconstruction and fusion network for multi-modal medical image fusion. On the one hand, this architecture parallels the reconstruction branch and the fusion branch which are linked by a shared encoder, thereby reducing error accumulation and improving the network’s feature extraction ability by multi-task learning. On the other hand, we have further constructed a feature decomposition network using parallel ViT and CNN modules to fuse base and detail features separately, while adding complementary links of high/low frequency information.

Experiments on three datasets demonstrate that our methods perform better than several typical traditional and DL-based image fusion algorithms in terms of eight fusion metrics and qualitative evaluations. Specifically, on multi-modal MR image fusion, our method produces fused images with excellent retention of bright details and smooth edge transition. For CT-MR image fusion, CIRF provides higher image contrast and better preservation of detail features from the original images. On SPECT-CT/MR image fusion, the fused images generated by CIRF are smoother while still retaining significant edge information. Furthermore, our method also exhibits strong generalization capability. In the future, we hope to extend our method to 3D medical image fusion.

## Figures and Tables

**Table 1:** Details of the three datasets.

Datasets	Image Pairs	Training	Testing
Atlas	Multi-modal MR	2976	62
	CT-MR	-	140
	SPECT-CT/MR	-	388
IXI	Multi-modal MR	3504	432
RIRE	CT-MR	2538	53

**Table 2:** The metric values of CIRF using different masking ratios (λ=0.3, σ=0.2) in Atlas Dataset.

Ratio	SD	PSNR	SCD	MI	SSIM	$Q^{\mathrm{AB}/F}$	VIFF	|rSFe|
0	92.705	24.709	1.769	1.478	0.889	0.549	0.679	0.143
0.1	91.686	24.975	1.761	1.483	0.885	0.539	0.673	0.230
0.2	92.980	26.700	1.758	1.456	0.743	0.502	0.671	0.252
0.3	91.946	26.698	1.749	1.458	0.862	0.500	0.662	0.308
0.4	93.808	27.799	1.736	1.441	0.442	0.460	0.658	0.311
0.5	92.734	29.311	1.733	1.460	0.854	0.494	0.658	0.320

**Table 3:** The metric values of CIRF using different λ (masking ratio = 0.1, σ=0.2) in Atlas Dataset.

λ	SD	PSNR	SCD	MI	SSIM	$Q^{\mathrm{AB}/F}$	VIFF	|rSFe|
0.1	83.998	18.678	1.632	1.489	0.894	0.555	0.638	0.256
0.2	89.120	21.384	1.732	1.466	0.880	0.531	0.660	0.232
0.3	91.686	24.975	1.761	1.483	0.885	0.539	0.673	0.230
0.4	94.165	23.402	1.778	1.443	0.787	0.491	0.674	0.208
0.5	97.534	28.796	1.790	1.458	0.779	0.488	0.684	0.207
0.6	97.995	27.097	1.790	1.438	0.764	0.451	0.682	0.202
0.7	100.142	26.650	1.802	1.437	0.753	0.436	0.686	0.184
0.8	100.008	23.018	1.796	1.418	0.724	0.395	0.675	0.217
0.9	104.269	25.418	1.787	1.422	0.411	0.358	0.677	0.185

**Table 4:** The metric values of CIRF using different σ (masking ratio = 0.1, λ=0.3) in Atlas Dataset.

σ	SD	PSNR	SCD	MI	SSIM	$Q^{\mathrm{AB}/F}$	VIFF	|rSFe|
0.1	92.022	25.153	1.761	1.461	0.878	0.519	0.670	0.243
0.2	91.686	24.975	1.761	1.483	0.885	0.539	0.673	0.230
0.3	91.863	24.283	1.761	1.476	0.867	0.537	0.676	0.210
0.4	92.272	23.768	1.768	1.466	0.806	0.523	0.675	0.223
0.5	92.088	24.461	1.762	1.473	0.782	0.534	0.674	0.210
0.6	91.952	26.342	1.758	1.493	0.887	0.544	0.673	0.218
0.7	90.754	23.280	1.751	1.490	0.884	0.536	0.670	0.227
0.8	94.179	27.839	1.767	1.465	0.846	0.529	0.677	0.217
0.9	90.745	23.266	1.755	1.481	0.841	0.536	0.667	0.242
1.0	89.141	21.417	1.747	1.487	0.887	0.535	0.667	0.239

**Table 5:** The metric values of CIRF using different masking ratio (λ=0.3, σ=0.2) in IXI Dataset.

Ratio	SD	PSNR	SCD	MI	SSIM	$Q^{\mathrm{AB}/F}$	VIFF	|rSFe|
0	72.361	39.814	1.872	2.347	0.744	0.740	0.981	0.047
0.1	72.122	36.206	1.809	2.251	0.709	0.727	0.945	0.160

**Table 6:** The metric values of CIRF using different λ (masking ratio = 0, σ=0.2) in IXI Dataset.

λ	SD	PSNR	SCD	MI	SSIM	$Q^{\mathrm{AB}/F}$	VIFF	|rSFe|
0.1	66.062	28.838	1.738	2.336	0.798	0.761	0.906	0.100
0.2	71.483	36.240	1.844	2.393	0.832	0.738	0.949	0.063
0.3	72.361	39.814	1.872	2.347	0.744	0.740	0.981	0.047
0.4	76.164	35.067	1.890	2.287	0.730	0.717	1.004	0.030
0.5	75.549	38.379	1.888	2.341	0.702	0.718	1.028	0.034
0.6	74.979	35.110	1.878	2.325	0.742	0.697	0.990	0.029
0.7	75.190	30.003	1.907	2.213	0.657	0.693	1.047	0.052
0.8	79.857	31.399	1.895	2.214	0.689	0.659	1.048	0.058
0.9	79.347	29.511	1.904	2.177	0.634	0.643	1.088	0.114

**Table 7:** The metric values of CIRF using different σ (masking ratio = 0.1, λ=0.3) in IXI Dataset.

σ	SD	PSNR	SCD	MI	SSIM	$Q^{\mathrm{AB}/F}$	VIFF	|rSFe|
0.1	72.160	34.644	1.889	2.336	0.730	0.733	1.004	0.033
0.2	72.361	39.814	1.872	2.347	0.744	0.740	0.981	0.047
0.3	70.964	32.695	1.870	2.315	0.734	0.735	0.970	0.033
0.4	72.028	30.786	1.876	2.223	0.683	0.728	0.993	0.033
0.5	70.364	31.237	1.854	2.278	0.715	0.731	0.974	0.040
0.6	72.816	33.506	1.881	2.288	0.713	0.735	0.991	0.036
0.7	73.270	33.861	1.877	2.269	0.710	0.728	0.990	0.032
0.8	72.297	29.842	1.873	2.221	0.680	0.733	0.978	0.044
0.9	69.995	28.954	1.847	2.213	0.671	0.732	0.955	0.056
1.0	73.257	33.528	1.878	2.272	0.694	0.728	0.993	0.031

**Table 8:** The metric values of CIRF using different masking ratios (λ=0.3, σ=0.2) in RIRE Dataset.

Ratio	SD	PSNR	SCD	MI	SSIM	$Q^{\mathrm{AB}/F}$	VIFF	|rSFe|
0	72.361	39.814	1.872	2.347	0.744	0.740	0.981	0.047
0.1	72.122	36.206	1.809	2.251	0.709	0.727	0.945	0.160

**Table 9:** The metric values of CIRF using different λ (masking ratio = 0.1, σ=0.2) in RIRE Dataset.

λ	SD	PSNR	SCD	MI	SSIM	$Q^{\mathrm{AB}/F}$	VIFF	|rSFe|
0.1	62.308	15.467	1.709	1.462	0.474	0.574	0.593	0.177
0.2	67.920	16.578	1.838	1.466	0.656	0.562	0.627	0.189
0.3	79.581	18.465	1.862	1.514	0.468	0.560	0.630	0.147
0.4	71.692	18.962	1.887	1.485	0.409	0.541	0.649	0.205
0.5	73.452	17.282	1.909	1.485	0.656	0.519	0.648	0.223
0.6	77.384	18.085	1.921	1.518	0.668	0.505	0.664	0.215
0.7	74.868	16.913	1.921	1.462	0.619	0.488	0.644	0.239
0.8	74.641	16.254	1.916	1.446	0.591	0.452	0.631	0.242
0.9	78.625	17.103	1.918	1.456	0.613	0.415	0.649	0.258

**Table 10:** The metric values of CIRF using different σ (masking ratio = 0.1, λ=0.3) in RIRE Dataset.

σ	SD	PSNR	SCD	MI	SSIM	$Q^{\mathrm{AB}/F}$	VIFF	|rSFe|
0.1	71.200	17.396	1.888	1.491	0.680	0.551	0.638	0.206
0.2	73.346	19.670	1.897	1.489	0.403	0.552	0.653	0.173
0.3	68.477	16.624	1.863	1.477	0.651	0.548	0.614	0.226
0.4	71.697	18.221	1.885	1.544	0.706	0.552	0.637	0.186
0.5	70.296	18.061	1.878	1.542	0.624	0.552	0.632	0.186
0.6	69.733	19.324	1.871	1.539	0.429	0.557	0.628	0.215
0.7	72.654	18.500	1.894	1.552	0.691	0.552	0.644	0.217
0.8	69.656	16.727	1.873	1.469	0.650	0.555	0.628	0.201
0.9	68.291	16.391	1.856	1.469	0.652	0.550	0.616	0.217
1.0	69.401	16.830	1.870	1.483	0.659	0.558	0.629	0.197

**Table 11:** The results of all computed algorithms on three datasets.

Methods	SD	PSNR	SCD	MI	SSIM	$Q^{\mathrm{AB}/F}$	VIFF	|rSFe|
CIRF	83.566 ± 11.21	25.520 ± 14.29	1.817 ± 0.06	1.628 ± 0.72	0.769 ± 0.30	0.585 ± 0.16	0.683 ± 0.30	0.179 ± 0.13
CDDFuse	71.144 ± 20.81	15.874 ± 10.47	1.412 ± 0.35	1.806 ± 0.61	0.837 ± 0.25	0.631 ± 0.14	0.593 ± 0.32	0.153 ± 0.12
U2Fusion	57.930 ± 27.62	3.913 ± 3.11	1.278 ± 0.13	1.677 ± 0.59	0.718 ± 0.31	0.488 ± 0.30	0.507 ± 0.23	0.389 ± 0.21
DenseFuse	53.216 ± 9.05	3.313 ± 3.27	1.339 ± 0.10	1.891 ± 0.60	0.772 ± 0.12	0.485 ± 0.32	0.499 ± 0.24	0.423 ± 0.23
IFCNN	61.115 ± 15.42	7.147 ± 5.89	1.363 ± 0.12	1.698 ± 0.61	0.857 ± 0.08	0.617 ± 0.19	0.565 ± 0.26	0.146 ± 0.29
NestFuse	66.902 ± 10.90	14.424 ± 8.09	1.480 ± 0.11	1.955 ± 0.68	0.857 ± 0.17	0.630 ± 0.16	0.599 ± 0.25	0.180 ± 0.16
RFN-Nest	63.489 ± 10.25	8.350 ± 7.44	1.558 ± 0.20	1.625 ± 0.53	0.700 ± 0.30	0.424 ± 0.26	0.550 ± 0.26	0.479 ± 0.24
PAPCNN	66.375 ± 19.16	12.104 ± 8.74	1.407 ± 0.14	1.588 ± 0.89	0.776 ± 0.14	0.542 ± 0.25	0.543 ± 0.31	0.251 ± 0.35
ReLP	70.915 ± 16.98	19.235 ± 8.04	1.338 ± 0.23	1.827 ± 0.81	0.869 ± 0.05	0.603 ± 0.14	0.589 ± 0.21	0.157 ± 0.08
TIF	84.423 ± 19.47	43.666 ± 24.94	1.708 ± 0.28	1.796 ± 0.58	0.781 ± 0.08	0.524 ± 0.13	0.672 ± 0.37	0.165 ± 0.11

## Data Availability

This study used open access online datasets [9,46,47].
